# Unmodified CdSe Quantum Dots Induce Elevation of Cytoplasmic Calcium Levels and Impairment of Functional Properties of Sodium Channels in Rat Primary Cultured Hippocampal Neurons

**DOI:** 10.1289/ehp.11225

**Published:** 2008-03-31

**Authors:** Mingliang Tang, Tairan Xing, Jie Zeng, Huili Wang, Chenchen Li, Shuting Yin, Dan Yan, Hongmin Deng, Jin Liu, Ming Wang, Jutao Chen, Di-Yun Ruan

**Affiliations:** 1 School of Life Science and; 2 Structure Research Laboratory, University of Science and Technology of China, Hefei, Anhui, People’s Republic of China

**Keywords:** calcium overload, cell viability, nanoparticles, QD, voltage-gated sodium channels

## Abstract

**Background:**

The growing applications of nanotechnologic products, such as quantum dots (QDs), increase the likelihood of exposure. Furthermore, their accumulation in the bioenvironment and retention in cells and tissues are arousing increasing worries about the potentially harmful side effects of these nanotechnologic products. Previous studies concerning QD cytotoxicity focused on the reactive oxygen species produced by QDs. Cellular calcium homeostasis dysregulation caused by QDs may be also responsible for QD cytotoxicity. Meanwhile the interference of QDs with voltage-gated sodium channel (VGSC) current (I_Na_) may lead to changes in electrical activity and worsen neurotoxicologic damage.

**Objective:**

We aimed to investigate the potential for neurotoxicity of cadmium selenium QDs in a hippocampal neuronal culture model, focusing on cytoplasmic calcium levels and VGSCs function.

**Methods:**

We used confocal laser scanning and standard whole-cell patch clamp techniques.

**Results:**

We found that *a*) QDs induced neuron death dose dependently; *b*) cytoplasmic calcium levels were elevated for an extended period by QD treatment, which was due to both extracellular calcium influx and internal calcium release from endoplasmic reticulum; and *c*) QD treatment enhanced activation and inactivation of I_Na_, prolonged the time course of activation, slowed I_Na_ recovery, and reduced the fraction of available VGSCs.

**Conclusion:**

Results in this study provide new insights into QD toxicology and reveal potential risks of their future applications in biology and medicine.

Quantum dots (QDs) are colloidal nanocrystalline semiconductors with unique optical and electrical properties (Bruchez et al. 1998). As a new class of inorganic fluorophore, which has the advantages of broad absorption spectra, narrow emission spectra, stable photostability, and long fluorescent lifetime, QDs are gaining widespread recognition and are rapidly applied to fluorescent labeling of cellular proteins (Kaul et al. 2003; Mansson et al. 2004; Sukhanova et al. 2004), cell tracking (Dubertret et al. 2002; Jaiswal et al. 2003), and even imaging *in vivo* (Akerman et al. 2002; Chen et al. 2004; Gao et al. 2004; Lim et al. 2003; Morgan et al. 2005). Although some reports have evaluated the cytotoxicity of various QDs in different cell lines under different circumstances (Chan et al. 2006; Kirchner et al. 2005; Lovric et al. 2005; Zhang et al. 2006), little is known about QD toxicity both *in vivo* and *in vitro*.

Santra et al. successfully labeled brain tissue with TAT-conjugated CdSratioMn/ZnS QDs that were intraarterially delivered to the rat brain (Santra et al. 2005), but this method of brain tissue labeling raised subsequent worries about QD toxicity on the toxin-susceptible brain. In fact, many QDs may seem harmless, but they can be destabilized because of their sequestration in tissues and long-term exposure to the bioenvironment. Cell structures and functions can be impaired when cells are exposed to unstable, poorly capped, or coated QDs (Choi et al. 2007, 2008; Hardman 2006; Lovric et al. 2005). In other words, even if the QDs are well modified, the potential risks are still present in subsequent biologic and clinical applications of QDs.

In the central nervous system (CNS), voltage-gated sodium channels (VGSCs) are responsible for both initiation and propagation of action potentials of the neurons. Therefore, potential modulation of the functional properties of VGSCs by QDs would be expected to alter the activity and functions of CNS neurons. Meanwhile, there is hardly a biological reaction in the CNS that is not regulated, directly or indirectly, by calcium ions. Transient rises of calcium ions in the cytoplasmic levels are believed to serve as second messenger signals that control numerous neuronal functions, whereas sustained elevation of cytoplasmic calcium levels is obviously deleterious to various neuronal functions. Of even greater concern is that the sustained increase of intra-cellular calcium may result in cell apoptosis/ death (Fox et al. 1999; McConkey and Orrenius 1996; Nicotera et al. 1994). Some reports have shown that QDs could impair cell functions and even induce cell apoptosis or death in certain cell lines (Chan et al. 2006; Choi et al. 2007, 2008; Medintz et al. 2005), but these studies mostly focused on the free Cd^2+^ (QD core degradation), free radical formation, and interaction of QDs with intracellular components, and little attention has been paid to the potential toxicity exerted by QDs through intracellular calcium steady-state and functional properties of neuronal ion channels.

Using confocal laser scanning and standard whole-cell patch clamp techniques, the present study explored the potential for the neurotoxicity of unmodified cadmium selenium (CdSe) QDs in a rat primary hippocampal culture model, focusing on cytoplasmic calcium levels and VGSC functions.

## Materials and Methods

### Cell culture and cell treatment

We used a hippocampal culture model in this study, as the hippocampus is the key learning and memory area of the brain. Low-density cultures of dissociated postnatal day 0 rat hippocampal neurons were prepared as described by Bi and Poo (1998) with some modifications. Hippocampi were removed from postnatal day 0 rat pups and treated with trypsin for 12–15 min at 37°C, followed by gentle trituration with a pipette. The dissociated cells were then plated at densities of 10^4^–10^5^ cells/mL on poly-l-lysine–coated glass coverslips in 35-mm Petri dishes or in 96/24-well culture plates (Costar, Cambridge, MA, USA). The plating medium was Dulbecco’s modified Eagle’s medium (DMEM) supplemented with 10% fetal bovine serum and 10% Ham’s F-12 with glutamine (all from Gibco, Carlsbad, CA, USA). Sixteen to twenty-four hours after plating, the culture medium was changed to a maintenance medium containing neurobasal media and 5% B-27 supplement (Gibco). Cultures were maintained at 37°C in a humidified atmosphere of 95% O_2_ and 5% CO_2_. Cultured neurons were used for experiments after culturing for 1 week.

For cell viability tests and electrophysiologic recordings, different volumes of 1-mM QD stock solutions dissolved in serum-free maintenance medium were added into 35-mm Petri dishes or 96/24-well culture plates (Costar) to the final desired concentrations of 1, 10, and 20 nM after cells were cultured for 1 week. The medium was replaced with standard maintenance medium after the cells were cultured in the QD-containing maintenance medium for an additional 24 hr before the experiments. For calcium imaging, different doses of QDs (1, 10, 20 nM) dissolved in the external solutions were micro-perfused onto individual cells through a quartz perfusion head.

Rats were obtained from the Laboratory Animal Center of the University of Science and Technology of China. All experiments were conducted in accordance with the National Institutes of Health *Guide for the Care and Use of Laboratory Animals* (Institute of Laboratory Animal Resources 1996). All efforts were made to minimize the number of animals used and their suffering.

### QD preparation

Unmodified CdSe QDs were synthesized and provided by J.-G. Hou and X.-B. Wang and co-workers (Structure Research Laboratory, University of Science and Technology of China). Additional details are available in their published report (Zeng et al. 2006). The CdSe nanoparticle was 2.38 nM in diameter. The freshly produced QDs were dialyzed for cadmium-free environment just before the experiments.

### DAPI staining

The apoptosis of cells was determined by evaluating nuclear condensation after staining cell nuclei with 4′,6-diamidino-2-phenylindole (DAPI) and by quantification of DNA fragment formation using Cell Death Detection ELISA^PLUS^ (Roche Molecular Biochemicals, Indianapolis, IN, USA). For DAPI staining, cells were washed two times with phosphate-buffered saline containing 137 mM NaCl, 2.7 mM KCl, 4.3 mM Na_2_HPO_4_, and 1.4 mM KH_2_PO_4_ (pH 7.2), fixed with 4% paraformaldehyde for 20 min, then incubated with 300 nM DAPI (Sigma Chemical Co., St. Louis, MO, USA). After labeling, cells were visualized using an Olympus microscope (Olympus, Tokyo, Japan) under light or filter designed for DAPI fluorescence. Cells were considered apoptotic when they showed either fragmented or condensed nuclei. At least 350 cells were counted in each experiment.

### MTT assay

The percentage of cell survival was measured using the 3-[4,5-dimethylthiazol-2-*yl*]-2,5-diphenyltetrazolium (MTT) colorimetric assay. MTT (0.5 mg/mL; Sigma) dissolved in the maintenance medium was added to the 96-well culture plates, and the cultures were incubated for an additional 4 hr at 37°C. The culture medium was then replaced with 200 μL dimethylsulfoxide (Sigma) to dissolve the formazan products in each well. Spectrophotometric data were measured using an ELX808 microplate reader (BioTeK, Winooski, VT, USA) at a wavelength of 570 nm. In each experiment, seven wells were used, and experiments were repeated three times.

### Calcium imaging

Primary cultured hippocampal neurons were washed with the standard external solution containing 150 mM NaCl, 5 mM KCl, 2 mM CaCl_2_, 1 mM MgCl_2_, 10 mM HEPES, and 10 mM D-glucose and buffered to pH 7.3. The standard external solution was continuously bubbled with carbogen (95% O_2_, 5% CO_2_). Cells were loaded with 5 μM fluo-3-AM (Molecular Probes, Eugene, OR, USA) and pluronic F-127 [Sigma, 0.004% (wt/vol) final] in the standard external solutions at 37°C for 40–45 min. Endogenous esterases converted nonfluorescent fluo-3-AM into fluorescent fluo-3. Cells were then washed twice with the external solutions and incubated at 37°C for 20–25 min before imaging. Cultures were imaged with a Carl Zesis scanning confocal microscope (Carl Zeiss Company, Heidenheim, Germany).

Cells were continuously perfused with the external solutions flowing at 5 mL/min, and different doses of QDs dissolved in the external solutions were microperfused onto individual cells through a quartz perfusion head. For calcium-free groups, calcium was excluded from the external solutions, while 40 μM EGTA (Sigma) was added to ensure calcium-free environment. For the thapsigargin group, cultures were incubated with 2 μM thapsigargin for at least 40 min before imaging to deplete endoplasmic reticulum (ER) calcium stores (Doutheil et al. 1999).

Epifluorescent excitation for fluo-3 was at 488 nm, and emission was collected at 510 nm. For measuring the change of cytoplasmic calcium levels under QD exposure, time-lapse sequences were recorded at a scanning rate of each 60 sec. Camera gain was adjusted to give baseline maximal fluorescence levels of 40–100 (arbitrary units) of a maximal 8-bit signal output of 256. Cell fluorescence (F) during the 5-min baseline period was F_0_. Fluorescence measurements for each cell were normalized to the average fluorescence intensity. Region of indexes (ROIs) were defined in the first image, and the normalized fluorescence changes (F–F_0_)/F_0_, that is, ΔF/F_0_, were measured throughout the image sequence. All settings of the scanning system and the complete data acquisition were controlled and collected by LSM 510 software (Carl Zeiss Company).

### Electrophysiologic recordings

Conventional whole-cell patch clamp recordings were performed in the primary cultured hippocampal neurons under standard configuration. Patch pipettes were pulled from glass capillaries with an outer diameter of 1.5 mm on a two-stage puller (PP-830, Narishige, Tokyo, Japan). The pipettes (3–5 MΩ resistance) were filled with a solution containing 120 mM CsCl, 20 mM tetraethylammonium chloride (TEA-Cl), 2 mM MgCl_2_, 10 mM EGTA, 2 mM ATP disodium (Na_2_-ATP), and 10 mM HEPES with pH adjusted to 7.20 using Tris-base and osmolality of 285–290 mOsm/L. Cells were in the standard external solutions 150 mM NaCl, 5 mM KCl, 2 mM CaCl_2_, 1 mM MgCl_2_, 10 mM HEPES, and 10 mM d-glucose. External solutions for recording VGSC current (I_Na_) containing 145 mM NaCl, 5 mM KCl, 1 mM MgCl_2_, 2 mM CaCl_2_, 0.2 mM CdCl_2_, 0.1 mM NiCl_2_, 5 4-aminopyridine (4-AP), 10 mM HEPES, and 10 mM glucose were applied onto the cells through the Y-tube microperfusion system (Murase et al. 1990). The pH of each external solution was adjusted to 7.2 with Tris-base and the osmolality was adjusted to 320–325 mOsm/L with sucrose; both external solutions were preoxygenated before use. Cells were considered only when the seal resistance was > 500 MΩ and the series resistance (< 30 MΩ) changed < 20% throughout the experiment. Fast and slow capacitances were neutralized, and series resistance was always compensated for > 85% with internal voltage-clamp circuitry.

### Data collection and analysis

Data were acquired with a PC-IIC patch-clamp amplifier (HUST-IBB, Wuhan, China) connected to a computer via an ITC-16 computer interface (Instrutech, Elmont, NY, USA), digitized at 20 kHz, filtered at 2 kHz, stored on a computer hard disk, and analyzed with Igor Pro 4.0 software (Wavemetrics, Lake Oswego, OR, USA) and Origin 7.5 (OriginLab Corporation, Northampton, MA, USA). Statistical analysis of the data was provided as mean ± SE. The effect of QDs on cell viability and cytoplasmic calcium levels was identified using a two-way analysis of variance, followed by a *post hoc* test. The effect of QDs on sodium channels was determined by using the Student’s *t*-test with paired comparisons. Significance for all values was set at *p* < 0.05.

## Results

### QDs could induce cell death in a dose-dependent manner

**Figure 1:**
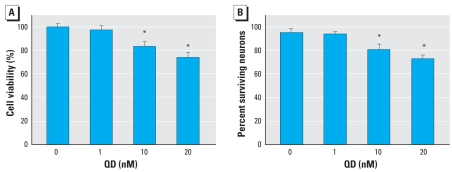
Effect of QDs on cell death in cultured control and QD-treated hippocampal neurons. (*A*) Cell viability measured by MTT assay; data represent mean ± SE of three independent experiments. (*B*) Percentage of surviving neurons (mean ± SE) evaluated using the DAPI staining method. *p < 0.05 compared with control.

We used the MTT assay and DAPI staining to test the viability of primary cultured hippocampal neurons under different doses of QD exposure for 24 hr. The percentage of cell survival was first measured using the MTT method. At 1 nM, QDs did not induce cell loss (97.6 ± 4.0% of control cells remained), but at high doses (10 and 20 nM), cell loss was significant compared with control (80.4 ± 4.3% and 71.2 ± 4.4% of control cells remained, respectively) (Figure 1A). Second, to determine the effect of QDs on cell viability in cultured hippocampal neurons, we used DAPI staining to assess the level of cell death caused by QDs. In accordance with the results above, we found no significant difference in this type of cell death between control cells and cells treated with 1 nM QDs (94.4 ± 3.6% survival vs. 92.8 ± 2.7%), whereas we did find significant differences between cells treated with 10 and 20 nM QDs and control cells (80.2 ± 4.8% and 72.1 ± 3.4% of surviving control cells, respectively) (Figure 1B). Both for the MTT assay and DAPI staining, we found significant differences when we compared 1-, 10-, and 20-nM QD groups with each other. These results demonstrated that QD treatment for 24 hr could increase the death of primary cultured hippocampal neurons dose dependently.

### QDs elevated intracellular calcium levels via extracellular calcium influx as well as internal calcium release

**Figure 2:**
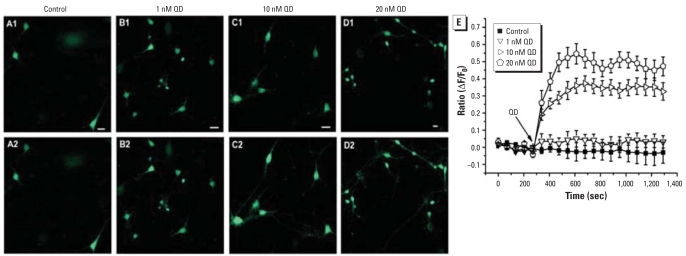
Effect of QD exposure on sustained elevation of cytoplasmic calcium concentration in cultured control QD-treated hippocampal neurons. In each experiment, the images were obtained under calcium fluorescence before QD exposure (*A1*–*D1*) and 5 min after QD exposure (*A2–D2*); bar = 20 μM. (*E*) Traces show mean ± SE of calcium fluorescence ratios (ΔF/F_0_) before and after QD exposure; 10 nM and 20 nM QD exposure significantly elevated calcium fluorescence.

We applied calcium imaging to assess the effect of QDs on calcium steady-state in cultured hippocampal neurons. It showed that acute 10- and 20-nM QD applications could increase the intracellular calcium levels as the Fluo-3 fluorescence ratio increased from basal to 0.33 ± 0.01 and 0.47 ± 0.02, respectively, which had a more significant increase compared with that of control (*p* < 0.01 and *p* < 0.01, respectively; *n =* 9), whereas acute 1-nM QD application failed to induce significant rise in intracellular calcium level compared with that of control (*p* > 0.05; *n =* 8) as the Fluo-3 fluorescence ratio changed to 0.03 ± 0.01 from basal. These data suggested that the acute application of QDs could affect intracellular calcium steady-state by elevating the intracellular calcium concentration (Figure 2).

To explore further that intracellular calcium elevation induced by QD application is due to extracellular calcium influx or internal calcium release, we used calcium-free external solutions and 2-μM thapsigargin preincubated external solutions.

**Figure 3:**
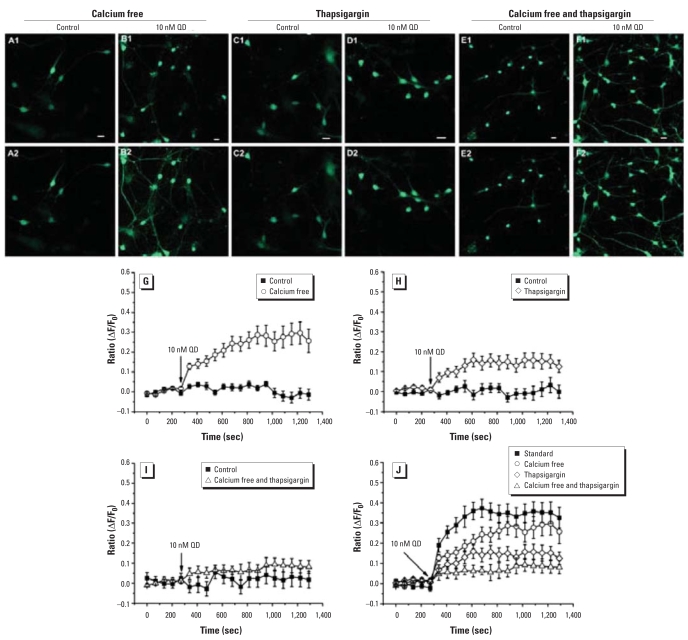
Extracellular calcium influx and internal calcium release involvement in QD-induced elevation of cytoplasmic calcium concentration in cultured hippocampal neurons. Images [(*A*; control) and (*B;* calcium free); (*C*; control) and (*D;* thapsigargin); (*E*; control) and (*F*; calcium free and thapsigargin)] were obtained under calcium fluorescence before QD exposure (*A1*–*F1*) and 5 min after 10 nM QD exposure (*A2*–*F2*). See “Materials and Methods” for details; bar = 20 μM. (*G–J*) Effect of 10 nM QD exposure on calcium fluorescence (ΔF/F_0_ ratio; mean ± SE) in (*G*) calcium-free external solutions, (*H*) thapsigargin-preincubated external solutions, (*I*) thapsigargin-preincubated calcium-free external solutions, and (*J*) standard external solutions, calcium-free external solutions, thapsigargin-preincubated external solutions, and thapsigargin-preincubated calcium-free external solutions.

Figure 3G shows that acute 10-nM QD application could elevate the Fluo-3 fluorescence ratio from basal to 0.23 ± 0.01 in the calcium-free group (*p* < 0.01 vs. control; *n =* 9) in cultured hippocampal neurons, whereas Figure 3H shows that acute 10-nM QD application could also induce a significant but smaller elevation on the Fluo-3 fluorescence ratio from basal to 0.13 ± 0.01 in the thapsigargin group (*p* < 0.01 vs. control; *n =* 9). When calcium-free solutions were pre-incubated with 2 μM thapsigargin, acute application of 10 nM QDs failed to significantly elevate the Fluo-3 fluorescence ratio, which was from basal to 0.07 ± 0.01 (*p* > 0.05 vs. control; *n =* 9) (Figure 3I). The extent of elevation of intracellular calcium levels induced by acute application of 10 nM QDs in standard external solutions, calcium-free external solutions, thapsigargin-preincubated external solutions, and thapsigargin-preincubated calcium-free external solutions is expressed in Figure 3J.

### Effects of QDs on VGSCs

Here we used a standard whole-cell patch clamp technique to study the effects of QDs on VGSCs in cultured hippocampal neurons. For recording inward sodium currents that were evoked by depolarizing pulses from the holding potential of −80 mV, voltage-gated potassium currents were blocked by intracellular Cs^+^ (120 mM) and TEA (20 mM) and extracellular 4-AP (5 mM); at the same time, voltage-gated calcium currents were extracellularly blocked by application of Cd^2+^ (0.2 mM) and Ni^2+^ (0.1 mM). The inward currents could be reversibly abolished by 0.5 μM tetrodotoxin (TTX) (data not shown), and thus were referred as TTX-sensitive I_Na_.

#### Effects of QDs on the properties of I_Na_ activation

**Figure 4:**
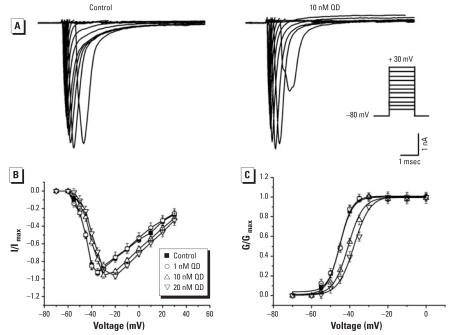
Effects of QDs on I_Na_ activation in cultured control and QD-treated hippocampal neurons. (*A*) Representative traces of activation of I_Na_ in control and 10nM QDs (I_Na_ was activated by a series of 50-msec voltage steps between −70 and +30 mV from the holding potential −80 mV; the increment was 10 mV except from −60 to −30 mV, where the increment was 5 mV). (*B*) Current-voltage relationships of I_Na_ in control, 1 nM QDs, 10 nM QDs, and 20 nM QDs (*n =* 8 per group); current amplitudes were normalized to the maximal I_Na_ peak value of each group. (*C*) Steady-state activation of I_Na_ in control, 1 nM QDs, 10 nM QDs, and 20 nM QDs (*n =* 8 per group). I_Na_ peak current values (I) were transformed into conductances (G) according to the equation G = I/(V_m_−V_rev_), where V_rev_ is the Na^+^ reversal potential and V_m_ is the membrane potential at which the current was recorded. The reversal potentials (V_rev_) were calculated from the crossing between the prolongation of the current-voltage curves and the horizontal axis: 55.7, 53.3, 54.3, and 56.5 mV, respectively, for control, 1 nM, 10 nM, and 20 nM QDs (not shown). Normalized peak conductances (G/G_max_) were fitted with a Boltzmann function G/G_max_= {1+exp[(V_1/2_−V_m_)/V_c_]}^−1^, where G_max_ is the maximal conductance; V_m_ is the command voltage; V_1/2_ is the potential of half-maximal activation; and V_c_ is proportional to the slope at V_1/2_.

Effects of QDs on the current-voltage curve (I-V) and on the conductance voltage (G-V) of I_Na_ are illustrated in Figure 4. We applied a series of 50-msec voltage steps between −70 mV and +30 mV preceded by the holding potential of −80 mV to evoke the currents. Representative traces of I_Na_ in control and in 10 nM QDs are presented in Figure 4A; 10 and 20 nM QDs induced depolarizing shifts of peak voltage, at which the current amplitudes reached the maximum, to approximately −30 mV and −20 mV, respectively. The peak voltage in 1 nM QDs was −35 mV, which showed no evident differences compared with the peak voltage in the control group (−35 mV) (Figure 4B).

The G-V curve was successfully fitted with a Boltzmann equation. The curves were shifted to the right in 10 and 20 nM QDs (Figure 4C). The values of V_1/2_, at which the conductance of I_Na_ reaches half of its maximum, were shifted from −45.3 ± 0.4 mV in control (*n =* 8) to −40.8 ± 0.5 mV in 10 nM QDs (*p* < 0.05 compared with control; *n =* 8) and −37.8 ± 0.7 mV in 20 nM QDs (*p* < 0.05 compared with control; *n =* 8). We found no significant difference between the values of V_1/2_ in 1 nM QDs (−45.1 ± 0.6 mV) and those in control (*p* > 0.05; *n =* 8).

#### Effects of QDs on time course of I_Na_

**Figure 5:**
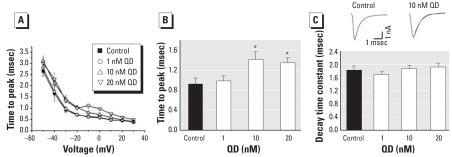
Effect of QDs on time course of I_Na_ in control QD-treated hippocampal neurons (*n =* 8 per group). (*A*) Time to peak of I_Na_ against the command voltage. (*B*) Time to peak at the command voltage of −30 mV plotted into histograms. (*C*) Time constants of fitted I_Na_ decay plotted into histograms. Insets are representative traces of I_Na_, whose decay phases were fitted with a single exponential function. *p < 0.05 compared with control.

The effects of different concentrations of QDs on time course of I_Na_, including its fast activation and rapid inactivation time, are presented in Figure 5. High concentrations of QDs post-poned activation of I_Na_ (Figure 5B) at the command voltage of −30 mV. In control cells, I_Na_ reached maximal peak 0.9 ± 0.1 msec (*n =* 8) after stimulation. In 1 nM QDs, I_Na_ reached maximal peak 1.0 ± 0.1 msec (*n =* 8), and it showed no significant difference compared with control. In cells incubated with 10 and 20 nM QDs, I_Na_ reached a maximal peak of 1.4 ± 0.2 msec (*n =* 8), and 1.3 ± 0.1 msec (*n =* 8), respectively, and both showed significant differences compared with control (*p* < 0.05). The effect of high concentrations of QDs on slowing down I_Na_ activation also existed at lower and higher voltages (Figure 5A).

The rapid inactivation phase of I_Na_, evoked by pulse from −80 mV to −30 mV, was well fitted with a single exponential equation and its time constant was calculated. In this study, QDs had no significant effect on the decay time of I_Na_ (Figure 5C). The decay time constants of I_Na_ were 1.8 ± 0.2, 1.7 ± 0.1, 1.9 ± 0.1, and 1.9 ± 0.2 msec in control (*n =* 8), 1 (*p* > 0.05 compared with control; *n =* 8), 10 nM QDs (*p* > 0.05 compared with control; *n =* 8), and 20 nM QDs, (*p* > 0.05 compared with control; *n =* 8), respectively.

#### Effects of QDs on properties of I_Na_ inactivation

**Figure 6:**
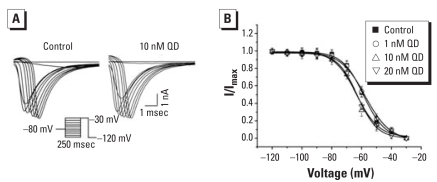
Effects of QDs on I_Na_ steady-state inactivation in cultured control and QD-treated hippocampal neurons. (*A*) Representative traces of steady-state inactivation of I_Na_ with 250-msec conditioning prepulses stepped from −120 to −30 mV and the membrane potential depolarized to a fixed test pulse of −30 mV to evoke inward I_Na_. (*B*) Normalized currents (I/I_max_) plotted against the voltages of conditioning pulses (*n =* 8) and fitted with a Boltzmann function I/I_max_= {1+exp[(V_1/2_–V_m_)/V_c_]}^−1^, where I_max_ is the maximal current, V_m_ is the conditioning voltage, V_1/2_ is the potential of half-maximal inactivation, and V_c_ is proportional to the slope at V_1/2_.

We examined effects of QDs on steady-state inactivation of I_Na_ using a double-pulse protocol; 250-msec conditioning prepulses, from −120 to −30 mV in 10-mV increments, were applied before step depolarization to the fixed potential of −30 mV (Figure 6A). As shown in Figure 6B, I_Na_ was normalized to the maximum current amplitude, and data were fitted with a Boltzmann equation; 10 and 20 nM QDs shifted the steady-state inactivation curves in the depolarizing direction. The value of V_1/2_ was −58.7 ± 1.4 mV in control (*n =* 8), significantly changed to −63.4 ± 1.7 mV and −62.8 ± 1.1 mV in 10 nM (*p* < 0.05 compared with control; *n =* 8) and 20 nM QDs (*p* < 0.05 compared with control; *n =* 8), respectively. We observed no significant change in 1 nM QDs (−57.5 ± 1.5 mV; *p* > 0.05; *n =* 8) compared with control.

#### QDs slowed I_Na_ recovery and reduced fraction of available sodium channels

**Figure 7:**
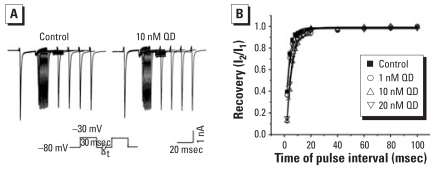
Effect of QDs on I_Na_ recovery in cultured control and QD-treated hippocampal neurons. (*A*) Representative traces of I_Na_ recovery. After a 30-msec conditioning pulse from the holding potential of −80 mV to −30 mV and a various interpulse interval of −80 mV ranging from 2 msec to 100 msec, a test pulse to −30 mV was subsequently applied. (*B*) Percentage of peak current recovery (I_2_/I_1_) against the time course of the interpulse interval (*n =* 8). The curves were well fitted with a single exponential function.

To study the time course of recovery of sodium channels from inactivation, we applied a double-pulse protocol as follows: a 30-msec conditioning pulse from the holding potential of −80 mV to −30 mV, a series of −80-mV intervals varying from 2 msec to 100 msec, and a −30-mV test pulse (Figure 7A). The peak value of I_Na_ evoked by the conditioning pulse was designated I_1_, whereas the peak value of I_Na_ evoked by the test pulse was designated I_2_. The ratio of I_2_ to I_1_ represents the recovery of sodium channels from inactivation. The plot of I_2_/I_1_ versus the duration of −80-mV intervals was well fitted with a single exponential equation and then its time constant was calculated. As shown in Figure 7B, the time constant of sodium channel recovery was 2.9 ± 0.3 msec in control (*n =* 8). It increased significantly to 3.6 ± 0.3 msec, 4.6 ± 0.2 msec, and 3.8 ± 0.2 msec in 1 (*p* < 0.0 compared with control; *n =* 8), 10 (*p* < 0.05 compared with control; *n =* 8), and 20 nM (*p* < 0.05 compared with control; *n =* 8) QDs, respectively. These results show that QDs significantly slowed the recovery of I_Na_ from inactivation.

**Figure 8:**
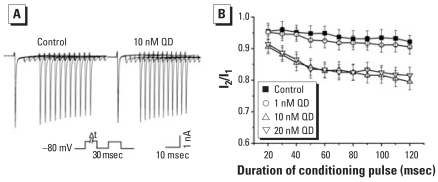
Effects of QDs on I_Na_ fraction of activated channels in cultured control and QD-treated hippocampal neurons. (*A*) Representative traces in control and 10 nM QDs; a conditioning pulse of various duration from 20 to 120 msec was first applied to modulate the level of Na^+^ channel inactivation and was followed by a 30-msec interval and subsequent test pulse. (*B*) Plots I_2_/I_1_ against the duration of the conditioning pulse (*n =* 8).

We also studied the effect of QDs on the fraction of available channels using a double-pulse protocol: holding potential of −80 mV, conditioning pulses to −30 mV with various durations ranging from 20 msec to 120 msec, 30-msec intervals of −80 mV, and then 50-msec test pulses to −30 mV. We regarded I_Na_ evoked by conditioning pulses and test pulses to −30 mV as I_1_ and I_2_, respectively. Along with the increase of the duration of the conditioning pulses, I_Na_ evoked by the test pulses decreased gradually (Figure 8A), indicating a larger fraction of inactivated channels (Cha et al. 1999). The ratios of I_2_/I_1_ were lower in 10 and 20 nM QDs than in control at all conditioning pulse durations; we observed no significant difference in 1 nM QDs compared with control (Figure 8B).

#### QDs had no effect on I_Na_ activity-dependent attenuation

**Figure 9:**
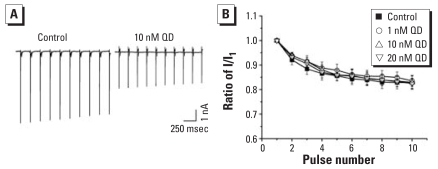
Effect of QDs on activity-dependent attenuation in cultured control and QD-treated hippocampal neurons. (*A*) Superimposed traces of Na^+^ channel attenuation in control and 10 nM QDs; a train of ten 30-msec depolarizing pulses to −30 mV were applied at the frequency of 5 Hz. (*B*) Normalized current amplitudes (I_2_/I_1_) were plotted against pulse number (*n =* 8).

We studied the effects of QDs on activity-dependent attenuation of sodium channels by applying a train of ten 30-msec depolarizing pulses to −30 mV at the frequency of 5 Hz. I_Na_ was gradually reduced with the sequence (Figure 9A), which may suggest the slow process of recovery of sodium channel from inactivation (Colbert and Johnston 1998). The ratios of the amplitude of the tenth to the first I_Na_ (I_10_/I_1_) were 0.84 ± 0.02, 0.83 ± 0.02, and 0.82 ± 0.02 in 1 nM QDs (*p* > 0.05 compared with control; *n =* 8), 10 nM QDs (*p* > 0.05 compared with control; *n =* 8), and 20 nM QDs (*p* > 0.05 compared with control; *n =* 8), respectively; the ratio was 0.83 ± 0.03 in control (*n =* 8) (Figure 9B).

## Discussion

With nanotechnology science developing, the prevalence of nanoparticles in society will be increasing, as will the likelihood of exposures. Many areas of these nanoparticles are unexplored, such as their potential adverse human health effects. Some scientists have tried to reduce QD toxicity for their further applications in the medical science through surface modifications, including conjugation and capping with biomolecules and polymers (Michalet et al. 2005; van Vlerken and Amiji 2006; Zhelev et al. 2006). The improved QDs may seem innocuous, but their sequestration in tissues or cells and long-term exposure to the bioenvironment can destabilize them, which further yields unprotected QDs. Unfortunately, the unprotected QDs can impair cell structures and functions and even induce cell death (Cho et al. 2007; Choi et al. 2007, 2008; Hardman 2006; Lovric et al. 2005). Labeling of brain tissues with QDs (Santra et al. 2005) and applications of QDs in the CNS may present inevasible, unprotected QD-induced risks on the toxin-susceptible CNS.

Impairment of cell structures and functions and a decrease in cell viability by QD treatment have been observed in a large number of *in vitro* studies in various cell lines, but few in neurons (Choi et al. 2007, 2008; Clarke et al. 2006; Kirchner et al. 2005; Medintz et al. 2005). In the present study, we found that QD treatment could increase cell death in primary cultured hippocampal neurons, which is consistent with previous studies noted above. When the cells were treated with QDs for 24 hr, certain morphologic changes such as shrinking of the plasma membranes and chromatin condensation in nuclei could be seen (data not shown); loss of plasma membrane integrity and chromatin condensation are considered events in necrosis and apoptosis. This is also consistent with the findings of Lovric et al. (2005), who reported that unmodified cadmium telluride QDs induced damage to plasma membrane, mitochondria, and nuclei. Further, QD-induced cellular damage can be partially prevented by *N*-acetylcysteine, a strong antioxidant containing a mercapto group, suggesting that QD-induced cyto-toxicity may be due in part to generation of reactive oxygen species. In fact, one possible mechanism postulated to be responsible for QD cytotoxicity is free radical formation, particularly reactive oxygen species (Clarke et al. 2006; Hoet et al. 2004; Oberdörster et al. 2005), during the synthesis and application of QDs. Because highly metabolically active mitochondria are particularly sensitive and vulnerable targets for cellular stress (Foster et al. 2006), previous studies focused attention on QD-induced impairment of mitochondria structures and functions. Overload of intracellular calcium concentration is also one of the main causes of cell death (Fox et al. 1999; McConkey and Orrenius 1996; Nicotera et al. 1994), and dysfunction of intra-cellular calcium homeostasis can be expected to impair various cell functions. In the present study, we focused on the potential QD-induced dysfunction of intracellular calcium homeostasis, and for the first time we found that unmodified QDs could rapidly and persistently elevate intracellular calcium concentration in cultured hippocampal neurons, which may have resulted in neuron death. The elevation of cytoplasmic calcium concentration is believed to be caused by the extracellular calcium influx and/or the internal calcium release from calcium stores, mainly in ER. When calcium was excluded from the external solutions, 10 nM QDs still induced a large elevation of cytoplasmic calcium concentration, but less than in standard external solutions. In cells preincubated with 2 μM thapsigargin, we observed a gentle increase in cytoplasmic calcium concentration, but the increase was much less than in standard solutions and calcium-free solutions. Taken together, we conclude that QDs can elevate the cytoplasmic calcium concentrations, in which both internal calcium stores (mainly ER) and extracellular calcium are involved; calcium release from internal calcium stores may be a main target of QD insult. Although previous studies showed that QDs bearing specific ligands could link to the given cell membrane proteins/receptors (Akerman et al. 2002; Dahan et al. 2003), nonspecific QDs could adhere to cell surfaces, possibly through interactions of QDs with glycoproteins and glycoplipids in the cell membrane (Hardman 2006). Several *in vitro* studies showed that QDs could be incorporated via endocytic mechanisms or receptor-mediated processes (Hoshino et al. 2004; Jaiswal et al. 2003). But how QDs trigger the extracellular calcium influx and internal calcium release from internal calcium stores is still not clear, and this is a new area that remains to be explored in understanding QD cytotoxicity.

Ion channels play an important role in cell viability and function, especially in the CNS, and their functional properties serve as a subtle indicator of the condition and viability of the cells. Kirchaner et al. (2005) found no impairment on the hERG channel and the inward rectified potassium channel in RBL and CHO cell lines treated by CdSe/ZnS under their experimental conditions. Considering that QDs may interact with various channels in plasma membrane, we selected the VGSC (a key molecular component responsible for both action potential generation and propagation) to examine the potential QD impairment on channel functions in primary cultured hippocampal neurons. The voltage dependence of the activation of sodium channels suggests that the transition from a resting, closed conformation to an open conformation is accompanied by the outward translocation of several positive charges across the membrane (Armstrong 1981; Hodgkin and Huxley 1952). In previous studies, the binding of β-scorpion toxins with the S3-S4 extracellular loop in domain α of the sodium channel α subunit enhanced closed-state inactivation, thus causing a left shift of steady-state inactivation (Cestele et al. 1998). Likewise, QD treatment caused a negative shift of steady-state inactivation, suggesting that the QD treatment shifts the sodium channel to the inactivated state and prevents its recovery from the inactivated state to the resting state, thus shifting the voltage dependence to a more negative membrane potential. As a result of the enhancement of inactivation, QD treatment in neurons also leads to a slowing of the recovery of sodium channels from inactivation and a reduction of the fraction of available channels. This coincidence suggests QDs or the degrading particles from QDs may bind to a receptor site in the S3-S4 loop at the extracellular end of the S4 segment in domain II of the α subunit. Considering that the S4 segments act as voltage sensors to initiate activation in response to changes in membrane potential (Catterall 1986; Stuhmer et al. 1989), the QD-induced changes in functional properties of sodium channels may be due to the binding of QDs or the degrading particles from QDs to some sites of S4 segments. At the same time, β-scorpion binding to neurotoxin receptor site 4 in the S3-S4 extracellular loop in domain α of the sodium channel α subunit was reported to shift the voltage dependence of activation to more negative potentials (Cahalan 1975; Jaimovich et al. 1982; Wang and Strichartz 1983). Paradoxically, our results were inconsistent with these results. In our observation, QD treatment induced a shift in the voltage dependence of activation of sodium channels in the depolarizing direction. The inconsistency between QD-induced impairment in kinetic characters of activation and inactivation of sodium channels implies the complexity and diversity of QDs or the degrading particles from QDs attacking sites in sodium channels. QD treatment also prolonged the time course of activation, and this observation is consistent with the elevatory threshold for activation. Binding of QDs or the degrading particles from QDs would lead to conformational changes of the channels, thus slowing the outward movement of the S4 extracellular loop in domain α of the sodium channel and delaying the opening of the channel. The modulation of VGSCs by QD treatment in primary cultured neurons may be one aspect of neuro-toxicity of unmodified QDs. However, the detailed mechanisms are still unknown, and further investigations are needed.

Although the mechanisms by which unmodified CdSe QDs elevated cytoplasmic calcium concentrations and impaired the functional properties of sodium channels in cultured hippocampal neurons remain a matter of conjecture, our results provide new insights into QD toxicology and present the potential risks of QD application, especially in the CNS, for scientific and clinical usage.
